# Supplementary material for: Imaging arbitrary incoherent source distributions with near quantum-limited resolution
Source: Sci Rep. 2022 Feb 18;12:2810. doi: 10.1038/s41598-022-06644-3 (PMC8857210; doi:10.1038/s41598-022-06644-3)
Supplement: Supplementary file 1 — Supplementary Information. [file 41598_2022_6644_MOESM1_ESM.pdf]

Supplementary Information for "Imaging arbitrary incoherent source distributions with near quantum-limited resolution."

Erik F. Matlin and Lucas J. Zipp

Applied Optics Laboratory, SRI International, Menlo Park, California 94025, USA

This document contains supporting material and details on (1) calculating the Quantum Cramér-Rao Bound, (2) a comparison to an imaging approach based on the eigenvectors of the multi-parameter SLD, and (3) details on the manifold optimization and adaptive imaging algorithm.

I. CALCULATING THE QUANTUM CRAMÉR-RAO BOUND

As noted in the main text, the Quantum Cramér-Rao bound (QCRB) is calculated from the inverse of the quantum Fisher information matrix $\mathcal{K}$ using the formula

$$\mathcal{K}_{kl} = \mathrm{Re}\left(\mathrm{tr}\left[L_k(\rho)L_l(\rho)\rho\right]\right), \tag{S1}$$

where $L_k(\rho)$ is the symmetric logarithmic derivative (SLD) of density matrix ρ computed as

$$\mathcal{L}_k(\rho) = \sum_{q,p;\lambda_q+\lambda_p\neq 0} \frac{2}{\lambda_q+\lambda_p}\langle e_q|\frac{\partial \rho}{\partial c_k}|e_p\rangle|e_q\rangle\langle e_p|, \tag{S2}$$

where λ_j and $|e_j\rangle$ are the eigenvalues and eigenvectors of ρ respectively.

The density matrix of the field at the image plane is given by

$$\rho = \int F(\boldsymbol{R})|\psi_{\boldsymbol{R}}^{PSF}\rangle\langle\psi_{\boldsymbol{R}}^{PSF}|\boldsymbol{dR}, \tag{S3}$$

and is computed directly in the discretized position basis. For our numerical calculations of the SLD, we truncate the sum in (S2) after the M largest eigenvalues, such that the results of the QCRB bound have converged to within a relative tolerance of 10^{-4}.

II. COMPARISON OF MO-SPADE IMAGING MODES TO THE EIGENVECTORS OF THE SLD

It is well-known that for single-parameter estimation problems, the QCRB can be attained by measuring in the basis corresponding to the eigenvectors of the SLD. In the multi-parameter estimation problem however, correlations between the parameters make it such that the SLD

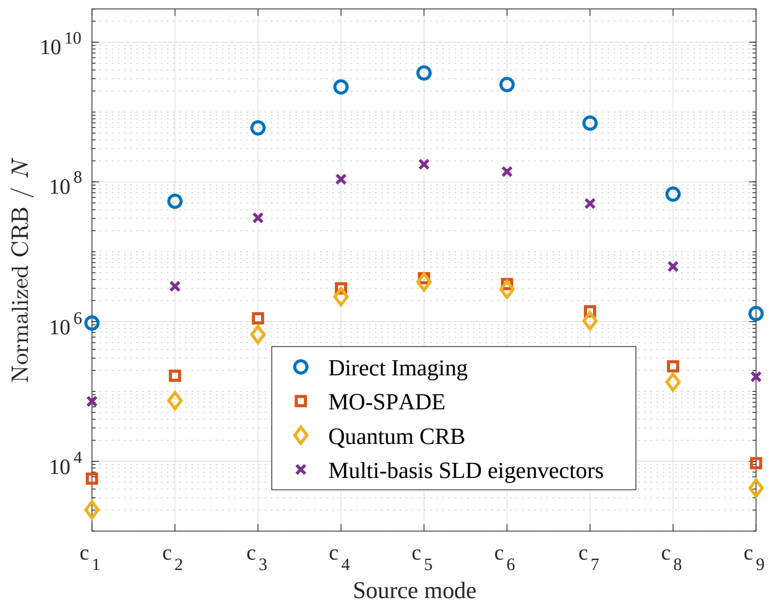

FIG. S1. Comparison to an SLD eigenvectors approach to imaging. The CRB values obtained for direct imaging (blue circles), the photon-splitting measurement into the K SLD eigenvector bases measurement (purple x's), and the manifold-optimized MO-SPADE imaging basis (red squares) are plotted for each source mode coefficient. The QCRB limit is also shown (yellow diamonds).

eigenvectors of a single parameter, while maximizing the Fisher information for that individual parameter, often result in an extremely poor classical Cramér-Rao bound (CRB) when the inverse of the Fisher information matrix is taken. Better results on the CRB can be achieved by assuming a measurement that splits the photons evenly between the K sets of SLD eigenvectors for all K source parameters. Figure S1 shows the resulting CRB of this multi-basis SLD eigenvectors approach compared with those of direct imaging, MO-SPADE imaging, and the QCRB for the 1D extended source of Fig. 3(b) in the main text, discretized into 9 rectangle modes. In this case, the CRB achieved by the multi-basis SLD eigenvectors approach, while an improvement over direct imaging, is still significantly worse than the CRB of the MO-SPADE imaging basis. It may be possible that knowledge of the SLD eigenvectors can be leveraged for selecting a suitable initial basis set for the manifold optimization algorithm, although we have not attempted this.

III. DETAILS ON MANIFOLD OPTIMIZATION

The manifold optimization algorithm, as described in the main text, seeks to find an orthonormal set of imaging modes $\Phi = \{\phi_j(\boldsymbol{r}) | j = 1, .., J\}$ that minimizes the objective function

$$L(\Phi) = \mathrm{tr}\left(W[\mathcal{I}(\boldsymbol{c};\Phi)]^{-1}\right), \tag{S4}$$

where W is a weighting matrix, and $[\mathcal{I}(\boldsymbol{c};\Phi)]^{-1}$ is the inverse of the Fisher information matrix. For a single measurement, the number of imaging modes J must be greater than or equal to the number of coefficients K in the source decomposition, in order to avoid an underdetermined source reconstruction. We have found empirically that a close approximation to the optimal imaging solution can usually be found when J is equal to K or $K+1$ for small numbers of modes. When optimizing over a large number of source parameters, including additional imaging modes can improve the convergence of the optimization routine. The imaging modes are discretized into P points and the objective function is minimized over all parameters using numerical manifold optimization techniques, specifically the Scaled Gradient Projection Method of Oviedo and Dalmau, as adapted from their Matlab code [1]. The approach requires the gradient of the objective function to be supplied, which can be calculated analytically or evaluated with automatic differentiation tools. For the 2D adaptive imaging problem, we developed a Python library that uses PyTorch's automatic differentiation and GPU capabilities to speed up the manifold optimization runtime.

IV. DETAILS ON ADAPTIVE IMAGING ALGORITHM AND COMPUTATION

The iterative adaptive imaging algorithm, fully described by the algorithm in Table I, consists of three main stages: imaging basis set optimization, photon detection, and source estimation. Since the source is presumed unknown, we have found that the most practical approach is to initiate the adaptive measurement process with a classical direct image, which corresponds to an imaging basis set of rectangular modes (ie. measuring in the image-plane position basis). After this first measurement is made, the initial source coefficient estimates $\hat{\boldsymbol{c}}_{\mathbf{0}}$ are found using an estimator g. After this initial estimate, the algorithm proceeds in a loop, with each iteration consisting of finding the optimal imaging basis set, performing a measurement in that basis, and then updating the source estimate.

An important part of the iterative algorithm is the inclusion of all previous measurement and basis information. For the source coefficient estimation, this means concatenating all measurements, concatenating all imaging modes, and weighting them by their relative photon contributions. Similarly, for the imaging mode optimization, we must modify the expression of the Fisher information to include the previous imaging modes, which are fixed, and the new imaging modes, which are to be optimized. The full expression for the Fisher information then becomes a convex combination of the Fisher information for both the previous imaging modes, $\Phi_{previous}$, and for the modes to be optimized, Φ_{new}, where their relative weight α is proportional to their relative photon budgets.

TABLE I. Algorithm for adaptive modal imaging.

1. Choose a set of source modes: $\{f_k(\boldsymbol{R})|k = 0 \dots K-1\}$.
2. Choose an initial set of J_0 orthogonal imaging modes: $\Phi_0 = \{\phi_j(\boldsymbol{r})|j = 0 \dots J_0 - 1\}$.
3. For $m = 0, .., M-1$:
   (a) Choose a measurement time for an expected number of photons, n_{ph}^m.
   (b) Perform measurement $y_m \sim n_{ph}^m [P(\phi_0^m), P(\phi_1^m), \dots, P(\phi_{J_m-1}^m)]^T$.
   (c) Estimate source coefficients $\hat{\boldsymbol{c}}_{\boldsymbol{m}} = g(\boldsymbol{y_0}, \dots, \boldsymbol{y_m}, \phi_0, \dots, \phi_m, n_{ph}^0, \dots, n_{ph}^m)$, where g is the estimator, e.g. non-negative least squares.
   (d) Calculate the estimated source: $\hat{F}_m(\boldsymbol{R}) = \sum_k \hat{c}_k^m f_k(\boldsymbol{R})$.
   (e) If $m < M - 1$:
      i. Estimate the Fisher information using all previous image modes: $\mathcal{I}_{est} = \mathcal{I}(\hat{\boldsymbol{c}}_{\boldsymbol{m}}, \Phi_0, \dots, \Phi_m)$.
      ii. Find the next set of orthonormal imaging modes by solving Eq. (S5).

The expression to be optimized is therefore:

$$\underset{\Phi_{new}}{\arg\min} \sum_k W[\mathcal{I}(\hat{\boldsymbol{c}}, \Phi_{previous}, \Phi_{new})]^{-1}_{kk}, \tag{S5}$$

where W is a weight matrix, the new imaging modes are constrained to be orthonormal $\langle \phi_i^{new}, \phi_j^{new} \rangle = \delta_{ij}$, and the updated Fisher information matrix is computed as

$$\mathcal{I}(\hat{\boldsymbol{c}}, \Phi_{previous}, \Phi_{new}) = \alpha \mathcal{I}(\hat{\boldsymbol{c}}, \Phi_{previous}) + (1 - \alpha)\mathcal{I}(\hat{\boldsymbol{c}}, \Phi_{new}). \tag{S6}$$

Finally, there is a question of the number of imaging modes to be used at each iteration. In our simulations, we have found that fixing the number of adaptive modes to be equal to the number of source modes works well, which is the strategy employed in producing both Fig. 5 of the main text. However, since the estimation problem then becomes over-constrained over multiple iterations, it is possible to achieve similar levels of performance with a reduced set of imaging modes, as seen in Fig. 4 which only uses 8 imaging modes. This is an area for future research.

As a further illustration of this methodology, a few of the imaging modes from the final adaptive step of the source measurement from Fig. 5 in the main text are shown in Fig. S2. In this case, the SGPM algorithm was initialized with an impulse basis, and signatures of this initialization may

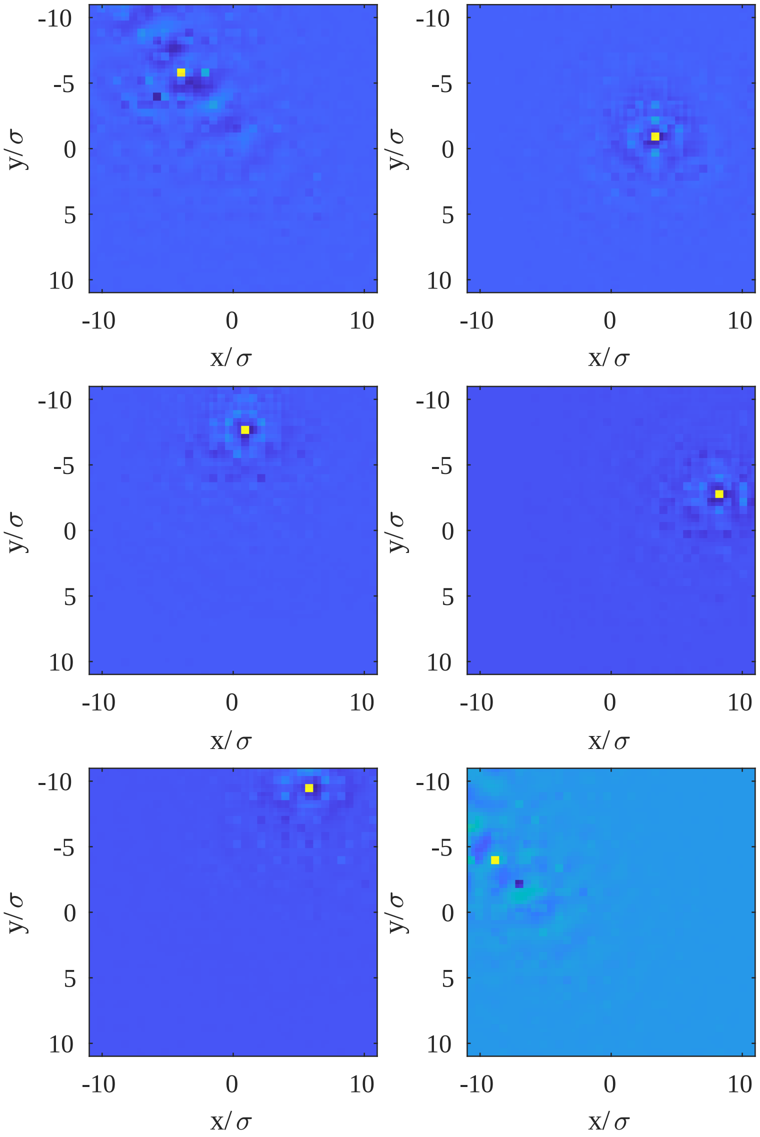

FIG. S2. Example imaging modes from the final adaptive step for the 2D source distribution of Fig. 5 in the main text.

still be present in the optimized basis set. Running the manifold optimization routine for more iterations may produce smoother imaging modes.

REFERENCES

[1] H. Oviedo and O. Dalmau, in *Advances in Soft Computing*, edited by L. Martínez-Villaseñor, I. Batyrshin, and A. Marín-Hernández (Springer International Publishing, Cham, 2019) pp. 239–250.
